# Automatic Fall Detection System Based on the Combined Use of a Smartphone and a Smartwatch

**DOI:** 10.1371/journal.pone.0140929

**Published:** 2015-11-11

**Authors:** Eduardo Casilari, Miguel A. Oviedo-Jiménez

**Affiliations:** Departamento de Tecnología Electrónica, Universidad de Málaga, Malaga, Spain; Cardiff University, UNITED KINGDOM

## Abstract

Due to their widespread popularity, decreasing costs, built-in sensors, computing power and communication capabilities, Android-based personal devices are being seen as an appealing technology for the deployment of wearable fall detection systems. In contrast with previous solutions in the existing literature, which are based on the performance of a single element (a smartphone), this paper proposes and evaluates a fall detection system that benefits from the detection performed by two popular personal devices: a smartphone and a smartwatch (both provided with an embedded accelerometer and a gyroscope). In the proposed architecture, a specific application in each component permanently tracks and analyses the patient’s movements. Diverse fall detection algorithms (commonly employed in the literature) were implemented in the developed Android apps to discriminate falls from the conventional activities of daily living of the patient. As a novelty, a fall is only assumed to have occurred if it is simultaneously and independently detected by the two Android devices (which can interact via Bluetooth communication). The system was systematically evaluated in an experimental testbed with actual test subjects simulating a set of falls and conventional movements associated with activities of daily living. The tests were repeated by varying the detection algorithm as well as the pre-defined mobility patterns executed by the subjects (i.e., the typology of the falls and non-fall movements). The proposed system was compared with the cases where only one device (the smartphone or the smartwatch) is considered to recognize and discriminate the falls. The obtained results show that the joint use of the two detection devices clearly increases the system’s capability to avoid false alarms or ‘false positives’ (those conventional movements misidentified as falls) while maintaining the effectiveness of the detection decisions (that is to say, without increasing the ratio of ‘false negatives’ or actual falls that remain undetected).

## Introduction

Unintentional injuries caused by falls among seniors are a major public health problem. According to different reports from the World Health Organization [1,2], a significant proportion (28%–35%) of the population over 64 suffers a fall per year. Direct medical costs associated with falls among American older people surpassed $20 billion in 2010 [3], an amount that is expected to reach $67.7 billion by 2020 [4].

A quick medical response after a fall occurrence has been proven to be key to reduce the morbidity and mortality of falls [5]. Therefore, the deployment of automatic, reliable and cost efficient Fall Detection Systems (FDS) has become a significant research topic during the last decade.

FDS, which can be regarded as an example of the application of Ambient Intelligence (AmI) paradigm to healthcare [6], can be classified into two general typologies [7]: context-aware and wearable systems. Context–Aware Systems (CAS), which include both vision-based and ambient-based architectures, are Wireless Sensor Area Networks [8] that analyze the signals captured from cameras, microphones and other environmental sensors [9], which are seamlessly placed around the patient to be tracked. CAS solutions present several drawbacks. Firstly, the zone where the patient is monitored is constrained to the specific area in which the sensors are installed. Uncontrollable circumstances in this area (changes in the illumination, noise, visual obstacles, falling objects, etc.) may alter the effectiveness of the detection. In addition, the setting up, tuning and maintenance of a CAS normally entail a non-negligible cost whereas the permanent visual observation of the system may compromise the patient’s sense of privacy. On the contrary, wearable FDS employ sensors (usually accelerometers) which are integrated in the clothes or transported by the patients as garments or personal gadgets. Wearable solutions directly measure physical variables describing the user’s movements without depending on the particularities of a restricted monitoring zone. In fact, if the transported devices incorporate wide area communication interfaces (e.g. a 3G/4G connection), the patient can be monitored almost in a ubiquitous way. Thus, wearable detection systems can be considered as a specific case of medical Body Area Networks (BANs) [10]. In this sense, wearable FDS can benefit from the computing capacities, embedded sensors and diversity of communication interfaces that are integrated in today’s smartphones. Smartphones are increasingly being proposed [11,12] as sensing and computing elements in cloud-assisted BANs aimed at tracking and processing the data flowing from body sensors both offline and online. In the area of fall detection, the rapidly declining prices and popularity of these devices have stimulated many research studies and projects proposing smartphone-based FDS over the last five years. In most cases, these proposals consist of ‘stand-alone’ architectures where the smartphone is the only element in the system, simultaneously acting as a sensor, communication gateway, alarming hardware and computing unit (to decide if a fall has occurred).

The chest and the waist have been proven [13] to be the best positions to place a wearable accelerometer aimed at detecting falls accurately, as they are typically close to the center of gravity of the human body. Some studies [14] have shown that the use of pockets to keep the smartphone diminishes the effectiveness of the detection procedure, as long as the device may move freely within the pocket and reduce the capability of the built-in accelerometer to characterize the mobility of the user. Thus, in some proposals in the literature, the detection systems yield optimal results only if the smartphone is fixed to these positions (chest or waist) with an adjustable band or a similar fixing element. However, this tight attachment of the phone clearly affects the patient’s comfort while hampering the freedom of using the conventional functions of the smartphone.

On the other hand, programmable commercial smartwatches, which can integrate accelerometers too, have also been proposed as an economical alternative to deploy wearable FDS [15–17]. When compared to smartphones, smartwatches improve the ergonomics of the system and (normally) the resolution and range of the employed built-in accelerometers. Conversely, the motion of the wrists (where the smartwatch is fastened) is not always representative of the body stability. So, sudden or abrupt movements of the arms that are not necessarily caused by falls may easily induce false positives (that is to say, activities that are misidentified as falls).

In order to reach a higher effectiveness and confidence of the fall detection decision, we propose a FDS that integrates two commercial Android devices: a smartphone and a smartwatch. Google’s Android is definitely the most extended Operating System (OS) for smartphones with a 82.8% market share during the second quarter of 2015 [18]. As a consequence, Android is being massively utilized as the programming environment for the development of mobile medical and social networks [19] and, in particular, of most smartphone-based fall detection systems that can be found in the literature [20].

The combined utilization of the accelerometry signals provided by the built-in sensor of the smartphone and an external (normally Bluetooth-enabled) accelerometer has been proposed in several previous studies such as [15,21,22]. However, the performance improvements introduced in the accuracy of the detection by the simultaneous use of the two sensors have not been systematically evaluated.

## Methods

**Fig 1:**
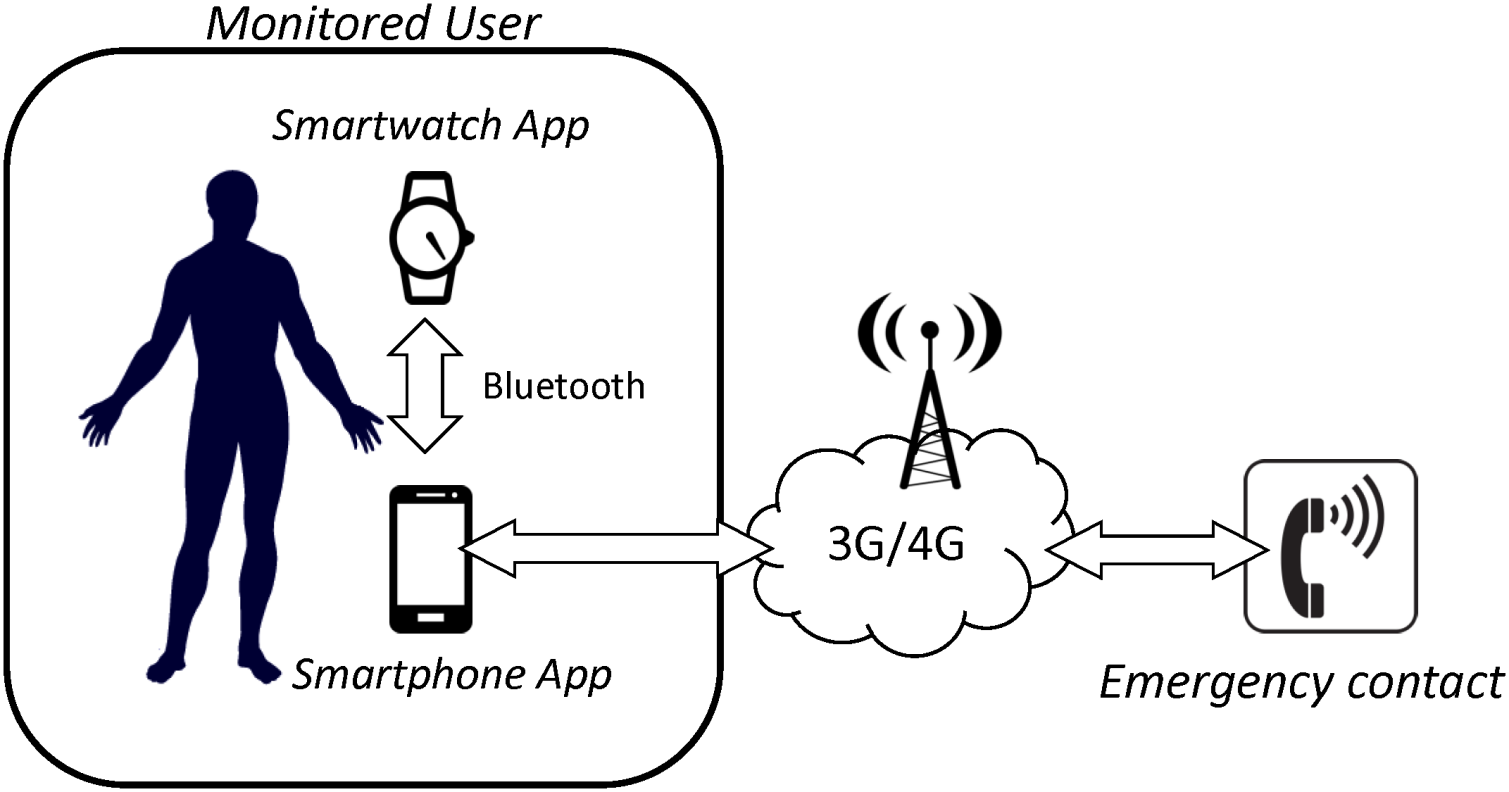
Basic Architecture of the Fall Detection System.

As aforementioned, the developed system, which is sketched in Fig 1, includes two basic elements: a smartwatch and a smartphone, both provided with Android Operating System and embedded mobility sensors (a triaxial accelerometer and a gyroscope).

The selected smartwatch was a LG W110 G Watch R model, with 1.2GHz Qualcomm Snapdragon 400 MSM8226 1.2 GHz processor, 512 MB of RAM, 410 mAh battery capacity and 4GB of internal storage. Although the system has also been tested with other smartphone models, the employed smartphone was a LG Nexus 5. This phone features a Qualcomm Snapdragon 800 2.26 GHz processor and 2 GB of RAM while it is powered by a 2300 mAh battery.

Each device carries out its own monitoring process independently. For that purpose, an Android application (*app*) was programmed to implement the different detection algorithms (which are described in the subsection 2.1) and installed in both devices. *Apps* are permanently tracking the user’s movements based on the data received from the accelerometer and the gyroscope that are embedded in the wearable devices.

The smartwatch and the smartphone incorporate short-range Bluetooth communications. Other wireless standards (802.15.4/ZigBee, 802.15.6 or Ultra-low power Wi-Fi) that are typically employed in healthcare BANs [23], are not considered because they are not commonly provided by commercial smartwatches or smartphones. The low scalability of Bluetooth networks (which may pose an important problem in other body area networks with multiple sensing motes) is irrelevant as the proposed network just consists of two nodes. Moreover, the Bluetooth specification natively implements different mechanisms at different layers (authentication, confidentiality, authorization, etc.) to guarantee secure communications, which is a crucial concern for the viability of any m-health (mobile health) sensor network [24–26] and medical BANs [10,27,28].

In the proposed system, as soon as a fall is detected by the *app* in the smartwatch an alerting message is transmitted to the smartphone via Bluetooth. A fall is only assumed to have occurred if the *app* running in the smartphone also detects a fall event within a short interval of 1 s before or after the reception of this message. In that case, a local acoustic alarm is triggered in the smartphone. If this local alarm is not deactivated by the user before 20 s, an automatic emergency call (or a text message) is sent to a preset contact phone number.

Accordingly, aiming at reducing the occurrence of false positives, the procedure for remote alerting is not initiated if the detection is only accomplished in a single device.

### Fall Detection Algorithms

The developed system allows comparing different fall detection techniques based on the analysis of accelerometer and gyroscope signals. As the computing and storage capacities in the smartphone and, especially, in the smartwatch, are limited, we discarded complex pattern recognition approaches (such as those based on artificial intelligence, rule-based or machine learning techniques) that have been proposed by the research literature (see [29] for a detailed state-of-the-art). Thus, we consider relatively simple ‘thresholding’ alternatives, for which a fall is detected only if one or several mobility variables exceed certain decision thresholds (in a simultaneous or consecutive way). In particular, we implemented the four following algorithms, which have been studied and compared by different works in the related literature [30]:

#### Basic Threshold Monitoring

As falls are associated to the presence of sudden peaks of the body acceleration, this method assumes that a fall has occurred if the module of the acceleration (or *SMV*, Signal Magnitude Vector) goes beyond a certain threshold (*SMV*_*Th*_). This module *SMV*_*i*_ (for the *i-th* measurement of the acceleration) can be computed as:

$$SMV_{i}=\sqrt{{\left|A_{x_{i}}\right|}^{2}+{\left|A_{y_{i}}\right|}^{2}+{\left|A_{z_{i}}\right|}^{2}}\;\;\;m/s^{2}$$ (1)

where *A*_*xi*_, *A*_*yi*_ and *A*_*zi*_ are the acceleration components, i.e., the readings in directions of *x*, *y*, and *z*-axis of the built-in tri-axial accelerometer of the smartphone or smartwatch for that *i-th* sample.

#### Fall Index

According to this algorithm, proposed by Yoshida in [31], a Fall Index (*FI*) is compared against a certain threshold (*FI*_*Th*_), instead of SMV. The *FI*_*i*_ index (for the *i-th* sample) can be estimated from the gradual variation of the last 20 measured values of the components of the acceleration in the x, y, and z-axis, as:

$$FI_{i}=\sqrt{\sum_{k=x,y,z}\sum_{i-19}^{i}{\left(A_{k_{i}}-A_{k_{i-1}}\right)}^{2}}$$ (2)

This method avoids the frequent false positives (induced by unexpected body movements), which take place when the basic threshold technique is applied. On the contrary, slow falls may remain unnoticed.

#### Two-phase detection

This technique, which is a variation of PerfallD algorithm presented in [30], bases its decision both on the *SMV* and the module of the acceleration at the absolute vertical direction (|*A*_*vi*_|), which can be calculated (for the *i-th* sample) as:

$$\left|A_{v_{i}}\right|=\left|A_{x_{i}}sin\theta_{z_{i}}+A_{y_{i}}sin\theta_{y_{i}}-A_{z_{i}}cos\theta_{y_{i}}cos\theta_{z_{i}}\right|$$ (3)

where *θ*_*yi*_ and *θ*_*zi*_ represent the measured values of the pitch and roll angles (for the *i-th* sampling interval), which can be sensed by the gyroscope integrated in the monitoring devices (smartphone and smartwatch).

The algorithm decomposes the analysis of the movements into two phases: free fall and impact. In order to detect the sharp decay of the acceleration caused by a Free Fall (*FF*), the algorithm permanently checks if the absolute maximum difference of the measured values of *SMV*_*i*_ within a certain (short) observation time window (*win*_*FF*_) exceeds a certain triggering threshold (*SMV*_*FF*_). If so, the recognition of the Impact Phase (*IP*) initiates. In this second phase, the devices compute again the difference between the maximum and minimum values of *SMV*_*i*_ within a second checking time window (*win*_*IP*_). If this difference surpasses another (higher) decision threshold (*SMV*_*IP*_), which may imply that an impact against the floor has occurred, a fall is suspected. A similar algorithm is applied in parallel to the variable *|A*_*vi*_ *|*, with the corresponding thresholds *AV*_*FF*_ and *AV*_*IP*_. A fall is only assumed if the two phases and the two detection conditions hold simultaneously for *SMV*_*i*_ and *|A*_*vi*_ *|*.

#### iFall

This procedure [32] also takes into account that a fall normally causes an initial abrupt decrease in the acceleration module. After this free-fall-phase, the shock on the floor provokes a brusque increase of the acceleration. So, a fall occurrence could be expected if the value of *SMV*_*i*_ goes beyond a lower (*SMV*_*l*_) and an upper threshold (*SMV*_*u*_) in the course of an observation interval (*win*_*O*_). However, a fall is only effectively detected if the patient actually moves from an upright posture to a horizontal position. For that goal, if the vertical position is not recovered within a second “post-fall” (*PF*) observation period (*win*_*PF*_), the detection is reported. In other case, the alarm is neglected.

## Results and Discussion

The proposed architecture was assessed with a series of systematic experiments. For that purpose, a set of falls and conventional movements (or Activities of Daily Living, ADLs) were simulated by 4 different volunteers (healthy males, aged between 22 and 29 years and 165–180 cm tall with an average weight of 67.5kg) in an indoor scenario (a domestic living room).

The experimental individuals simulated three types of falls (forward, lateral and backward falls). On the other hand, ADLs consisted of three categories of ordinary mobility patterns: walking, standing from sitting (and vice versa) and others (comprising movements such as making gestures with the arms, turning, running or answering the phone). Experiments were iterated ten times per subject for every type of fall and ADL and for every considered detection algorithm. For all the tests the smartwatch was worn on the right wrist while the smartphone was located within a trouser pocket (next to the thigh of the right leg).

In many experiments described by the literature, the smartphone is attached to the chest or the waist in order to obtain a better characterization of the human mobility. The use of a second detecting device in our architecture avoids these ‘unnatural’ positions of the smartphone (which could cause discomfort to the patient).

All the experiments were repeated to compare the performance of the system with the cases in which the detection is merely based on the individual decisions made by the smartwatch or the smartphone separately.

In order to judge the effectiveness of the systems to discriminate falls from ADLs, after observing the response of the system to the different mobility patterns, we computed the amount of false negatives (i.e., actual falls that were not detected by the system) and false positives (ADLs that were mistakenly recognized as falls) in the tests. From these two measurements (*FN* and *FP*) we estimated the values of the *sensitivity* and *specificity*, two metrics that are commonly employed by the literature to evaluate the efficacy of FDS and, in general, the performance of systems for pattern recognition with binary classification

These two metrics (which respectively describe the capability of the system to identify falls and ADLs properly) are defined (as percentages) as follows:

$$Sensitivity\;=100\cdot \frac{TP}{FN+TP}\;\%$$ (4)

$$Specificity\;=100\cdot \frac{TN}{FP+TN}\;\%$$ (5)

where *FN* and *FP* indicate the numbers of false positives and false negatives while *TP* (True Positives) and *TN* (True negatives) indicate the numbers of actual falls and ADLs that have been correctly classified, respectively.

The decision thresholds of the algorithms were selected based upon the results of an initial ‘tuning’ test phase (prior to the final evaluation), aiming at achieving a reasonable trade-off between the incidence of false positives and false negatives. In particular, we set the following values for the thresholds and observation intervals required for the four detection algorithms: *SMV*_*Th*_ = 25 m/s^2^ (for basic thresholding), *FI*_*Th*_ = 46 m/s^2^ (for *Fall Index* algorithm), *win*_*FF*_ = 0.1 s, *win*_*IP*_ = 1 s, *SMV*_*FF*_ = 7.5 m/s^2^, *SMV*_*IP*_ = 18.5 m/s^2^ *AV*_*FF*_ = 6.5 m/s^2^ and *AV*_*IP*_ = 16.5 m/s^2^ (for the two-phase detection), *win*_*O*_ = 1 s, *win*_*PF*_ = 20 s, *SMV*_*l*_ = 2.5 m/s^2^ and *SMV*_*u*_ = 24 m/s^2^ (for *iFall* algorithm)

The results of the experiments for the four contemplated algorithms and the different types of falls and ADL are summarized in Table 1. This table describes the system performance for the case in which the fall detection requires a simultaneous identification of the fall in both the smartphone and the smartwatch. On the contrary, Tables 2 and 3 portray the same results when just the decision made by the smartphone or the smartwatch is taken into account to detect the fall.

**Table 1:** Combination of smartphone and smartwatch.

	Sensitivity				Specificity			
	Type of Fall				Type of ADL			
Algorithm	Forwards	Backwards	Lateral	Global	Walk	Sit/Stand	Other	Global
**Basic Threshold**	85.0%	70.0%	80.0%	78.3%	100.0%	100.0%	80.0%	93.3%
**Fall Index**	95.0%	90.0%	90.0%	91.7%	100.0%	95.0%	80.0%	91.7%
**Two-phase**	95.0%	90.0%	90.0%	91.7%	100.0%	100.0%	100.0%	100.0%
**iFall**	100.0%	95.0%	95.0%	96.7%	100.0%	100.0%	95.0%	98.3%

**Table 2:** Results using only the smartphone to detect the falls.

	Sensitivity				Specificity			
	Type of Fall				Type of ADL			
Algorithm	Forwards	Backwards	Lateral	Global	Walk	Sit/Stand	Other	Global
**Basic Threshold**	100.0%	100.0%	90.0%	96.7%	100.0%	90.0%	60.0%	83.3%
**Fall Index**	80.0%	100.0%	100.0%	93.3%	100.0%	100.0%	60.0%	86.7%
**Two-phase**	100.0%	90.0%	90.0%	93.3%	90.0%	100.0%	80.0%	90.0%
**iFall**	90.0%	90.0%	100.0%	93.3%	100.0%	100.0%	80.0%	93.3%

**Table 3:** Results using only the smartwatch to detect the falls.

	Sensitivity				Specificity			
	Type of Fall				Type of ADL			
Algorithm	Forwards	Backwards	Lateral	Global	Walk	Sit/Stand	Other	Global
**Basic Threshold**	100.0%	90.0%	80.0%	90.0%	100.0%	100.0%	70.0%	90.0%
**Fall Index**	90.0%	100.0%	100.0%	96.7%	100.0%	80.0%	60.0%	80.0%
**Two-phase**	100.0%	90.0%	100.0%	96.7%	100.0%	100.0%	80.0%	93.3%
**iFall**	100.0%	100.0%	90.0%	96.7%	90.0%	100.0%	80.0%	90.0%

Tables show that the combined use of the smartphone and the smartwatch improves the specificity of the system in the range of 5–15% for the four analyzed algorithms. This can be explained by the fact that false positives detected by one device are neutralized by the correct identification of the other Android device. This improvement is achieved just at the cost of a slow decrease in the sensitivity (except for the case of using the basic threshold algorithm, which is too simplistic to provide an accurate simultaneous detection in both devices). The combined solution also enables a more homogeneous and reliable behavior of the detector when we analyze the typology of the tested ADL. For instance, the results in Table 2 (for the solution that only considers the detection in the smartphone) and 3 (for the case where the fall decision is only based on the smartwatch) indicate that a poor specificity of 60% is achieved by two algorithms in the case of testing the system under a variety of ADLs (group of movements described under the term ‘Other’). As Table 1 illustrates, this deficient performance is clearly enhanced by the combined scheme.

Another important point that is frequently disregarded by the literature is the way in which these two performance metrics (sensitivity and, especially, specificity) must be analyzed from a practical point of view. For example, a value for the specificity higher than 95% (which implies that 95% of actual ADLs are properly recognized) might be initially considered as an indicator of an acceptable system operation. However, this number entails that (as an average) one out of every twenty ADLs (e.g. sitting, run, walking stairs, etc.) will generate a ‘false positive’ (i.e., a false alarm). Thus, depending on the patient’s mobility, false alarms can be triggered very often, which can be particularly annoying for the patient (if he/she has to manually disable the emission of emergency calls or alert messages) or the remote monitoring user (who can be misinformed). We investigated this situation with our system by evaluating the number of false positives detected after tracking one of the experimental users during 24 hours of daily life. Table 4 shows that for the four algorithms (even using the combined solution that takes into account the detection on the smartphone and the smartwatch) several false positives took place.

**Table 4:** Number of false positives detected after 24 hours of continuous monitoring.

Algorithm	No. of false positives
**Basic Threshold**	5
**Fall Index**	4
**Two-phase**	2
**iFall**	4

Another crucial aspect when dealing with an application running on an Android device is power consumption. The permanent computation of the algorithm or the constant readings of the embedded sensors may exhaust the batteries in the wearable devices, rendering the monitoring system infeasible in practice.

**Fig 2:**
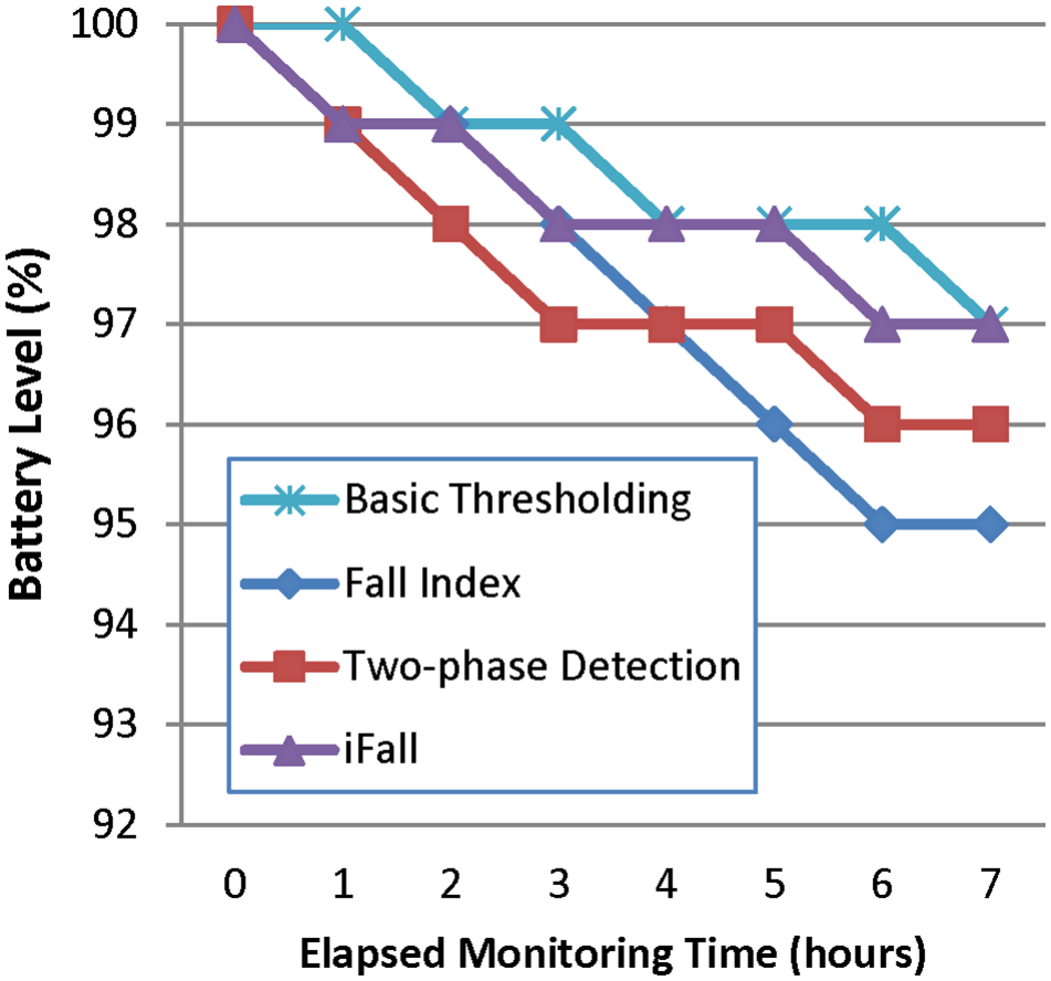
Evolution of the battery level in the smartphone.

**Fig 3:**
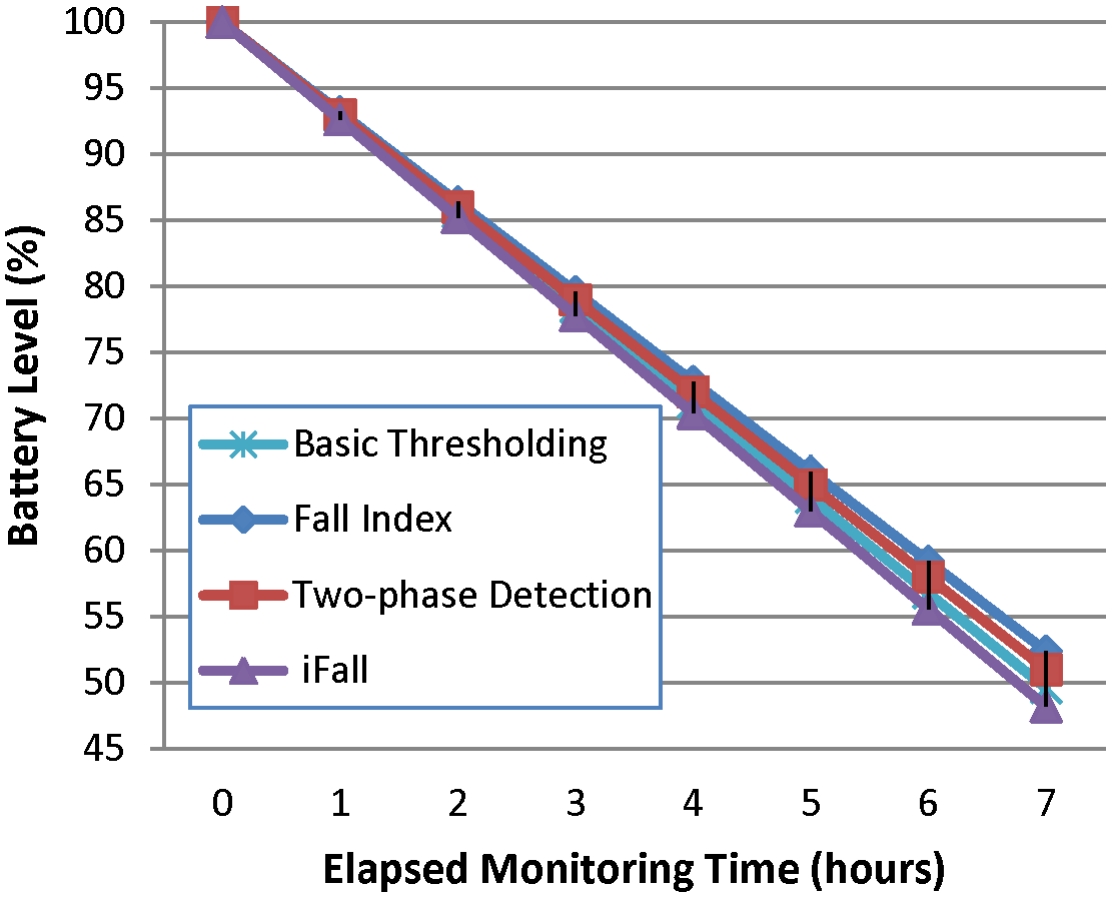
Evolution of the battery level in the smartwatch.

We have analyzed the evolution of the battery level in the smartphone (Fig 2) and the smartwatch (Fig 3) as a function of the operating time of the system. For each analysis, the batteries are initially fully charged and then their status is periodically checked as the monitoring process progresses. No other *app* was run in the devices during the measurements. Fig 2 shows that the impact of the FDS on the power capacity of the smartphone is not critical (although not negligible) as long as after 7 hours, the battery level is still over 95%. Contrariwise, the power drain in the smartwatch is much more evident (see Fig 3), as more than 50% of the battery capacity is depleted after the same period of 7 hours. In our opinion a viable FDS should utilize devices with a minimum autonomy of 24 hours, so that they can be recharged at least during the patient’s sleep. In any case, results indicate that the election of the detection algorithm is almost irrelevant in terms of consumption.

The usage of memory and computing resources (CPU) by the fall detection application in the Android devices was also proved to be moderate (60 MB of memory) or negligible (less than 2% of CPU load) in the tested smartphone models. So, no serious ‘coexistence’ problems with other running apps may be expected.

Other relevant issues for the social acceptance of a wearable monitoring system are usability and ergonomics. The usability of the application was judged as satisfactory by the test subjects. Similarly, the ergonomics of the system was also positively evaluated by the interviewed users as watches and smartphones are already present in the their daily life. Hence they do not introduce any new, intrusive, bulky, wearable component. Moreover, other proposals of smartphone-based FDS require the smartphone to be worn tightly attached to the chest or the waist in order to increase the precision in the fall detection decision. In our scheme, the detection relies on two elements, so that the smartphone can be transported in a more natural way (e.g. in a pocket). In any case, further studies on usability, ergonomics and acceptance among older users are underway to improve the system.

Our proposal has been evaluated through a set of falls simulated by young adults. Due to the evident difficulties (or risks) of assessing the performance of the detection systems by analyzing real body falls of older people, this is the test procedure massively employed by the literature on fall detection systems. In this field, it is still a controversial issue if the falls mimicked by healthy young people can be judged as representative of the actual falls that elderly experience [33].

In [34] authors contrasted the mobility patterns obtained from simulated falls and those falls suffered by a set of older adults who were monitored during a period six-month. From the comparison authors conclude that there are significant similarities between the real-life falls of older people and the falls emulated by middle-aged individuals.

## Conclusions

The utilization of the inherent sensing and computing capabilities of Android personal devices, which are currently omnipresent in the daily life of the citizens, has fostered the deployment of cost-effective Android-based systems for automatic fall detection.

This paper has described and evaluated a prototype of a wearable fall detection system which benefits from the joint analysis of the user mobility with two commercial Android devices: a smartphone and a smartwatch. In contrast with most proposals in the literature, which are based on the motion sensors of a single device, the system only considers that a fall has occurred if it is simultaneously detected by both devices (which can seamlessly intercommunicate through a Bluetooth connection). The system allows choosing between four different detection algorithms based on simple thresholding techniques proposed in the literature. Results obtained with systematic tests and a set of simulated falls and Activities of Daily Living show that the combined use of both devices clearly increase the capability of the system to discriminate ADLs (specificity) just at a the cost of a small decrease in the efficiency to identify falls (sensitivity). The actual importance of a certain value of the specificity is assessed by tracking an experimental user during a long period. Results indicate that the occurrence of recurrent false alarms may reduce the attractiveness of these wearable solutions for fall detection. Finally, the paper also shows that the limited battery of Android devices may hinder their use in these systems.
